# Extracts from a Private Letter from Prof. Freer, Dated Stuttgart, Sept. 13, 1867

**Published:** 1867-11

**Authors:** J. W. Freer, J. Adams Allen


### Foreign Correspondence.

### EXTRACTS FROM A PRIVATE LETTER FROM PROF. FREER, DATED STUTTGART, SEPT. 13th, 1867.

DEAR DOCTOR:

The International Medical Congress assembled, according to programme, on the 16th of August, A.D. 1867, at the old L'Ecole de Médicin. At least 700 or 800 delegates were present at the opening exercises. The hall is miserable for such purposes—shaped very much like a well-developed clamshell. The officers and privileged characters occupied the hinge part, while the audience were packed, in what was left, on benches without backs, and dirty as an American college near the close of a winter session. Prof. Bouillaud, in his little welcoming address, acknowledged themselves apprentices in the art of entertaining their medical brethren, at least, compared with their friends over the water. I would not have disputed him on any account, for they could, at least, in that great city have obtained a better hall. Nevertheless, most of the members seemed to take things good-naturedly, and tucking their bifurcated coat-tails under their arms, patiently committed their noble bottoms to the merciless embrace of unyielding oaken planks, in order to witness rare equestrian feats of old and practiced "hobby-horse riders." These exhibitors have become so skilled, and have their horses so well in hand, that they can sail between the narrow boundaries which serve to separate "tweedle-dee from tweedle-dum," without grazing a stirrup. A Broadway omnibus-driver would have been struck dumb with admiration and envy. *Vide* the papers and discussions on the anatomy, physiology, and pathology of tubercle. The transactions will be published *in extenso*, undoubtedly, when, most likely, one may be able to make some profitable gleanings.

The question of proposing to the various civilized governments some efficacious measures for restraining the propagation of venereal maladies, excited sharp debates, and much ill-natured criticism. There occurred some very lively repartee on the value and propriety of syphilization, between Ricord and Auzeas Turenne, the latter being a firm advocate of its efficacy, but has contented his zeal by practising upon others instead of himself, which is looked upon by Ricord as the *experimentum crucis* of a man's faith in his doctrines. Whether this prophylactic measure has any intrinsic merit or not, it certainly received very little favor at the hands of the Congress, and the spirit manifested by the opposition was rather that of prejudice than fair dealing with a weighty scientific problem, but its advocates seem to be possessed of the true martyr spirit, and bared their devoted heads to the showers of ridicule and ill-natured criticism, with an air of resignation which would have been creditable to the persecuted saints of olden time.

By the way, Ricord appears and acts enough like Governor Evans, formerly Professor of Obstetrics in Rush Medical College, to be taken for a twin brother. This is no disparagement to the Governor, for I count myself as one of his admirers.

You will find in those numbers of the *Gazette des Hopitaux* that I have sent you, two papers on the subject of preserving anatomical specimens, which are, probably, of much value. I saw a preparation by the Phenique acid method, which was perfectly pliable, and plump as a recent dissection, even the small joints of the fingers were as flexible as in the most recent state, and at the same time, the specimen—which was a well-dissected arm—was as dry as leather, and without odor, other than from the acid, which is not at all disagreeable. This is something worth looking into—if the acid is not too expensive, the method is invaluable for medical colleges. The process by tannic acid must be very tedious and expensive.

Our French hosts gave one entertainment, as you will observe by the toasts and speeches which you will find reported. I did not attend, on account of a slight indisposition.

I visited the hospitals, Hotel Dieu, Pitie, and others. In the first named, is the celebrated Maisonnueve, whom I followed through a morning clinic. He is, as you are probably aware, outside the faculty, having no other position than his hospital appointment; but this is enough, and just suits his genius, for being a progressive as well as "fighting man," it gives him an opportunity to deal his blows impartially, right and left, and he does not fail to embrace the opportunity, by animadversion and trenchant criticism on the doings and teachings of his confréres. His figure, features, and physiognomy reminds one of an educated, demonstrative Irishman.

In Paris, Phenique acid is, at present, king over all other remedial agents, and, as a surgical dressing, is particularly so with Maisonneuve. Charpie, saturated with a solution of this acid, is applied to every breach of continuity of surface by this doughty professor, and with the assurance in his teachings that it obviates nearly all the secondary surgical accidents. A tin wash-dish containing the inevitable embrocation, is a conspicuous object at every bedside, and by the side a small stack of charpie, from which, as he arrives, he snatches a handful, dabs it in the solution, and, with a vigorous hand, slashes it on to every variety of diseased or abraded surface, from vulva to remotest parts. He does not attempt union by "first intention," and this is true of all the Paris surgeons at the present time. It is taught that the open wound is less likely to lead to purulent infection and kindred surgical accidents. Hotel Dieu is an immense structure, and so well kept that it is the very paragon of neatness and order.

At the Pitie, we find Désormeaux. His clinics are well attended and very popular. His hobby is, as you are aware, the endoscope. I had the pleasure of looking through it many times, as he is very obliging, and takes great pleasure in giving ample explanations of the method of using it. In his hands, it certainly performs all that has been claimed, and reveals the bladder and urinary passages wonderfully well. It must soon be an indispensable appendage to every surgeon's armament.

Desmarres' eye clinic is a clinical curiosity rather than a means of instruction. I will not attempt to estimate the numbers in waiting, but during an hour's time, he disposed of at least one per minute. His secretary was obliged to write a race in order to keep pace with his off-hand declarations of diagnosis, prognosis, treatment, etc. Those requiring the use of the ophthalmoscope are turned off to an adjoining room, for future consideration.

Laskowski is the rising man in this department of surgery. Although, as yet, the number of patients is rather limited, yet his clinic is very valuable, because of his zeal and ability as a teacher. His ophthalmoscope is, probably, the most perfect and convenient in use. The most unskilful can use it with perfect facility.

I started one morning, bright and early, to pay a visit to the venerable Velpeau's clinic, but, alas, he was dead (the news of which must have reached you long since). His funeral took place that very morning, so I attended that instead of the clinic. The ceremonies were of the most imposing character, for he died laden with honors and riches. "*Requiescat in pace.*"

Velpeau's museum occupied considerable of my time, as I was not only deeply interested in the specimens of pathology, but wished to learn something of the method of getting up and arranging such affairs. Much of it is simple and easy of accomplishment.

I am under special obligations to my friend, Dr. Bosworth, for much that I saw, as he has been on the ground several years, and is, consequently, well posted. I will have occasion to speak of him again on my return. I saw —— ———, "formerly of Michigan," at the Congress, but did not have a chance to meet him, as he occupied the opposite side of the hall. He looked very happy and motherly, cheered when others did, and listened as attentively as though he understood it all.

By the way, Bosworth has promised to write for our journal, you may expect a paper for your next number. He is liberally educated and thoroughly posted in the profession, so I think you will get something from him to your liking. He has been a most faithful follower of the clinics in Paris these three years, as shown by his documents and notes.

Regarding the sanitary arrangements of Paris, we might take valuable lessons in some things, and they of us in others. We, of them, respecting urinals in public places, for every prominent object in this great city is considered a proper mark for a suffering bladder. A Frenchman can no more forbear wetting everything that projects than an American dog, and that irrespective of time, place, and surroundings. This arrangement gives a delightful odor of ammonia, which, you know, is an antidote for fainting; and I have noticed that the ladies do not faint there, even at the sight of these mictatorial exploits.

Another arrangement, for the protection and bestowal of unclaimed babies, may be worth copying. These consist of little round structures, situated on the public streets, with a porthole-like arrangement on one side, as a place of deposit. The shelf which recieves the "waif" appertains to a revolving arrangement on the inner side, which is at once turned by an unseen hand half around, where it (the baby) is received and transported to the hospital for foundlings. This arrangement became necessary for the prevention of the crime of infanticide. As the depositor is never seen, this method must address itself to them as preferable to child-murder. I need not mention the legalization of prostitution, as another sanitary arrangement.

I am afraid I have already written more than you will have the patience to read, so please present my regards to the Faculty, and other friends. Fraternally yours,

J. W. FREER.

Prof. J. ADAMS ALLEN.

